# Supplementary material for: Are carbon emissions trading and green financial instruments synergistic? -Comprehensive quantitative research based on content analysis
Source: PLoS One. 2024 Mar 7;19(3):e0298601. doi: 10.1371/journal.pone.0298601 (PMC10919601; doi:10.1371/journal.pone.0298601)
Supplement: S1 Data — (ZIP) [file pone.0298601.s001.zip › Data/地级市Z_F工作报告.zip!/╕╩╦α/╜≡▓2╩╨/└┤╫╘╣1⁄2╓┌║┼í╛┬φ┐╦╩2╛▌═°í┐_2016.docx]

各位代表：

      现在，我代表市人民政府向大会作工作报告，请予审议，并请各位政协委员和列席会议的同志提出意见。
　　**一、“十二五”及2015年工作回顾**
　　“十二五”时期，面对“三期叠加”阵痛持续加深、多重困难矛盾相互交织、改革转型任务日益繁重的严峻形势，在省委、省政府和市委的正确领导下，在市人大、市政协的监督支持下，我们深入贯彻落实党的十八大、十八届三中、四中、五中全会及习近平总书记系列重要讲话精神，紧紧扭住全面建成小康社会奋斗目标，在攻坚克难上勇于担当，在补齐短板上主动作为，在干事创业上凝心聚力，基本完成了“十二五”规划确定的各项目标任务。
　　——综合经济实力大幅提升。生产总值年均增长11.6%，人均生产总值达到47739元；累计完成固定资产投资1013亿元，年均增长23.1%；社会消费品零售总额年均增长14%；一般公共预算收入年均增长12.6%；城镇居民人均可支配收入、农村居民人均可支配收入年均增长10.9%、14%。这五年是全市上下迎难而上、砥砺前行、励精图治的五年，是转型步伐加快、社会全面进步、城乡面貌发生巨大变化的五年，也是改革不断深化、民生持续改善、人民群众得到实惠最多的五年。
　　——结构调整成效明显。三次产业结构不断优化，由2010年的5.3：79.3：15.4调整为8：58.2：33.8。有色金属新材料、化工循环、新能源及装备制造“三大重点产业”加快发展，有色金属就地加工转化率达到58%，镍网生产能力占全国的55%，培育形成了10条循环经济产业链，建成全省首个百万千瓦级光伏发电基地，风光电并网规模达到210万千瓦，我市被确定为全国有色金属新材料战略性新兴产业区域集聚发展试点地区、国家新能源示范城市和甘肃省新能源电力就地消纳试点城市，循环经济“金昌模式”入选国家区域循环经济典型案例。金川集团公司荣获“中国工业大奖”,国家级镍钴新材料实验室挂牌成立。现代农业快速发展，永昌县、金川区现代农业示范区创建为省级示范区，清河现代循环农业产业园、金昌现代畜牧产业园等9个产业园创建为省级示范园，农村专业合作组织达到1181个，认定家庭农场226个。文化旅游产业突破发展，“紫金花城•神秘骊靬”大景区建设加快推进，成功举办三届骊靬文化国际旅游节，《骊靬情》《花语》等有创造力、传播力、影响力的文化产品进一步提高了金昌美誉度和知名度。骊靬古城、紫金花城、金水湖景区晋升为4A级景区，打造出紫金赏花、骊靬揭秘、祁连揽胜、工矿探奇、乡村寻美、沙漠历险等六大旅游名片，文化及相关产业增加值占GDP比重比“十一五”末提高了2.1倍，旅游综合收入年均增长46.1%。
　　——城乡统筹协调发展。国家新型城镇化综合试点、金川区双湾镇国家建制镇示范试点、永昌县城基础设施投融资体制改革试点工作全面推进，城乡一体化模式被列为全国典型案例，常住人口城镇化率达到67.96%。公共服务均等化水平不断提高，新建城市道路49.54公里，建成农村集中居住点76个，改造农村危房15648户，开工建设各类保障性住房及实施棚户区改造33273套(户)，广播电视、行政村等级公路、农村人饮安全实现全覆盖。金水新区、龙首新区建设加快推进，金阿高速、金阿铁路竣工验收，金武高速建成通车，金昌机场建成通航，热电联产及供热管网、天然气入户等一批公共服务设施和城市街景改造、龙首湖公园、龙泉景观带二三期等一批城市景观工程顺利建成。城镇管理水平不断提升，数字化城管、社区网格化精细化管理体系日益完善，群众精神文明创建活动成效显著，双湾镇、水源镇北地村入选第四届全国文明村镇，我市成功创建为国家卫生城市、全国文明城市，第二次荣获全国未成年人思想道德建设工作先进城市称号。
　　——生态文明加快推进。生态环境持续改善，大力实施祁连山生态保护、石羊河流域综合治理等重点生态工程，森林覆盖率达到24.27%，建成区绿化覆盖率达到37.6%，建成南坝乡、双湾镇2个国家级生态乡镇和焦家庄乡、宁远堡镇新华村等16个省级生态乡镇(村)，累计建成市级、省级美丽乡村示范村12个，完成70个行政村环境连片综合整治，新城子镇油菜花景观被评为“中国美丽田园”，我市成功创建为国家园林城市。节能减排成效显著，深入实施环境保护重点工程，化学需氧量、氨氮、二氧化硫、氮氧化物排放量分别比2010年下降6.5%、13%、6.68%、16.77%。金川集团公司累计投入40多亿元，实施“蓝天碧水”等系列工程，二氧化硫回收利用率达到96%以上，市区环境空气中二氧化硫浓度年均值达到二级标准，预计单位生产总值能耗累计下降25.8%，万元工业增加值用水量下降16.36%，我市被列为国家第二批低碳试点城市。资源综合利用水平不断提高，全国工业固废综合利用示范基地通过省上审核，工业固废综合利用率提高到75%。城市生活垃圾无害化处理率达到100%，秸杆综合利用率达到82%，废旧农膜回收利用率达到72%，规模畜禽养殖废弃物利用率达到87%。
　　——改革开放深入推进。经济体制、文化体制、社会体制、生态文明体制改革取得重要进展，理顺了金昌国家级经济技术开发区管理体制和运行机制，合并成立了市文广新局和市卫计委，工商、质监移交地方管理，组建了农艺研究院，医药卫生体制改革和公立医院改革稳步推进。国资国企改革全面启动，金川集团公司总校及所属中小学移交地方管理，金泥集团公司股份制改革、八冶建设集团公司发展混合所有制和员工持股试点等工作积极推进。35家企业在甘肃股权交易中心实现托管，31家企业挂牌交易。农村综合配套改革深入推进，水权转换机制进一步规范，土地确权试点及集体林权制度改革任务全部完成，土地流转率达到58.4%。认真贯彻落实新预算法，全口径预算管理、部门预算管理、国库集中支付等各项改革深入推进。兰州银行、甘肃银行等金融机构入驻金昌，组建交通、文化旅游、开发区等投融资公司3家。建立了市级PPP项目库，列入国家和省级推荐项目11个。开通北京、西安、兰州等5条航线，金昌海关及出入境检验检疫局开展报关报检业务，全市外贸企业达到95家。
　　——人民群众幸福指数不断提高。坚持“双联”行动与精准扶贫融合推进，建立了“双联”帮扶基金和救助基金，扶贫对象人均纯收入年均增长15%以上，贫困人口减少2.75万人。城乡医保、低保、养老水平大幅提高并在全省率先并轨运行，就业、就学、培训、公共卫生服务、住房等实现均等化。文化事业快速发展，我市成功创建为全国首批国家公共文化服务体系示范区，金昌大剧院、市图书馆、县文化中心、乡村舞台、金昌文化街等一批文化基础设施建成投用，市博物馆、区“三馆”基本建成，农家书屋、农村电影放映等文化惠民工程实现全覆盖。有色冶金职业技术学院办学质量不断提升，市理工中专迁建工程全面完成，市五中开工建设，累计投入32亿元实施了中小学校安工程、农村薄弱学校改造工程等项目，全市义务教育均衡发展率先通过国家评估认定。市人民医院迁建、市第一人民医院迁建、市消毒供应中心等项目顺利推进，全市二级以上医疗机构床位使用率增长22.1%，传染病发病率较“十一五”末下降50%以上，公立基层医疗机构全部实行基本药物零差率销售。建成体育惠民工程37个，“一村一场”126个，体育场地达到1212个，人均体育场地面积高于全国平均水平，连续四年成功举办全国青少年户外体育活动营地夏令营暨中国•金昌国际青少年生存训练营活动，成功举办市第七届运动会，竞技体育水平不断提高。获准设立国家重点实验室1家、省级工程技术研究中心6家，培育农业科技示范点125个，应用类科技成果转化率达到36%，科技进步贡献率由“十一五”末的53%提高到57%，我市被确定为省级创新型试点城市。审计、统计、气象、人防、档案、地方志、国防动员、国土资源、防震减灾、妇女儿童、民族宗教、住房公积金等各项工作都有了新的发展，我市被命名为全国残疾人工作示范城市，双拥模范城“七连冠”通过省级验收。
　　——民主法制建设切实加强。自觉接受人大依法监督和政协民主监督，人大代表意见建议、政协委员提案办复率达到100%。修订完善政府工作规则和重大事项决策程序，社会听证、社会公示和专家咨询、法律顾问等制度体系不断完善，科学决策、民主决策、依法决策机制逐步健全。简政放权步伐加快，市级具有行政审批事项的部门由42个减少为37个，行政审批事项由457项缩减为217项，减幅达52.5%，总工作时限压缩46%。认真贯彻落实中央八项规定精神和省委“双十条”、市委“十项规定”，扎实开展党的群众路线教育实践活动和“三严三实”专题教育，政府机关办事效率和服务水平进一步提高。全面落实党风廉政“一岗双责”制度，反腐倡廉取得新的成效。深入推进“法治金昌”建设，“六五”普法任务全面完成。扎实推进“平安金昌”建设，加强政府应急管理，完善矛盾纠纷排查调处机制，健全社会治安防控体系，人民群众的安全感明显增强。
　　各位代表！2015年，我们全面落实稳增长、促改革、调结构、惠民生、防风险的政策措施，及时研究出台促进经济稳定增长的30条工作措施，千方百计促进经济社会平稳健康发展。实现生产总值224.5亿元，同比增长3.2%；完成固定资产投资248.6亿元，增长0.6%；大口径财政收入44.1亿元，下降9%；一般公共预算收入18.5亿元，增长2.7%；社会消费品零售总额76亿元，增长8.6%；城镇居民人均可支配收入达到29670元，增长10.4%；农村居民人均可支配收入达到11459元，增长11.2%。
　　一是项目建设扎实推进。全市确定的70个重点项目开工率96.7%，争取国家专项建设基金9.77亿元、政府债券7.66亿元；实施招商引资项目338项，到位资金246.83亿元，同比增长22%；“引大济西”工程、兰州至张掖铁路三四线、铁路现代物流中心、“紫金云”大数据产业园、韩家峡水库等重大项目前期工作积极推进，市养老康复中心开工建设。
　　二是工业经济转型升级步伐加快。40万吨离子膜烧碱二期、30万吨PVC、镍铜矿伴生铂族贵金属高效回收与综合利用等项目开始试车，铜阳极泥综合利用等项目进入设备安装调试阶段，铜尾矿综合利用一期、超细金属及氧化物试验生产线等项目完成厂房主体建设。“一企一策”研究解决企业生产经营中的困难和问题，新能源就地消纳试点工作成效明显，新能源企业新增发电量4.2亿千瓦时，让利工业企业1.13亿元。
　　三是农业农村经济稳步发展。以高原蔬菜、优质肉羊、优质苜蓿、食用菌为主的优势特色产业进一步发展壮大，畜禽饲养量达到245万头(只)，市级以上农业产业化龙头企业达到51家，完成高效节水灌溉面积50.23万亩，我市被确定为全省农产品质量安全市，永昌县被确定为首批创建国家农产品质量安全试点县。全面落实“1+9”精准脱贫方案，贫困人口减少6000人。大力发展劳务经济，输转城乡富余劳动力8.4万人次，创劳务收入14.88亿元。
　　四是第三产业快速发展。安排财政专项资金1.4亿元撬动旅游业发展，“紫金花城•神秘骊靬”大景区建设性规划和文化产业发展规划编制完成，成功举办首届香草美食文化旅游节暨“薰衣草之约”集体婚礼，紫金苑、北部绿色生态景观区、花文化博览馆等项目加快建设，文化旅游产业在第三产业中的占比达到23.1%。大力发展现代服务业，发展电子商务平台23家，开设网店300多家，电子商务销售额超过1亿元。银行业金融机构各项贷款余额397.07亿元，同比增长67.94%。
　　五是城镇规划建设管理水平不断提高。“数字城管”二期设计方案编制完成，永昌县“多规合一”试点工作加快推进。新改建市区道路5条6.23公里，市行政中心、市污水处理工程升级改造等项目建成即将投用。治脏、治乱、治差、治污、治堵等“五治专项行动”取得明显成效，国家“智慧城市”加快推进，国家卫生城市通过复审验收。
　　六是改革创新有序推进。大力推进大众创业、万众创新，行政审批制度改革深入推进，市县(区)“三张清单一张网”初步建成，政府服务社会经济发展能力不断提高。财税体制改革不断深化，“营改增”试点有序推进，“三证合一、一照一码”工商登记制度全面实施，新增非公市场主体4068户，预计非公经济增加值占GDP的比重达到28.5%。
　　七是民生保障力度加大。省列10项27件民生实事和市列“八大民生工程”19件实事全面完成，城镇登记失业率2.95%，开工建设保障性住房11165套，完成农村危房改造3660户，建成城市社区日间照料中心6个、农村老人幸福互助院34个。深入推进社会治理“五大专项行动”，社会大局和谐稳定。
　　各位代表，过去五年的成绩来之不易，为今后发展奠定了坚实基础，这是省委、省政府和市委正确决策、坚强领导的结果，是市人大、市政协共谋发展、监督支持的结果，是全市人民和衷共济、地企密切合作、军政团结奋斗的结果。在此，我代表市人民政府，向所有关心、支持和参与全市经济社会发展的各界人士和同志们，表示衷心的感谢！
　　各位代表，过去五年的经验弥足珍贵，为推动金昌发展积累了宝贵财富，我们必须坚持发展第一要务不动摇，主动适应新常态，全面落实五大发展理念；必须坚持经济结构调整不动摇，不断做优一产、做精二产、做大三产；必须坚持统筹城乡发展不动摇，促进区域之间、城乡之间协调发展；必须坚持狠抓项目建设不动摇，把实施“3341”项目工程作为既利当前、更惠长远的重要抓手；必须坚持增进民生福祉不动摇，努力使全市老百姓享受到人有普保、学有良教、病有适医、老有颐养、住有安居的幸福生活；必须坚持凝心汇智聚力不动摇，珍惜长期形成的四大班子精诚团结、地企军地密切协作、全市上下和衷共济的良好局面。
　　各位代表，过去五年也积累和凸显了不少困难问题，我们必须直面应对、着力破解。一是主要经济指标年均增速没有实现规划预期目标，地区生产总值、固定资产投资和进出口贸易总额等指标没有完成2015年度计划目标，第三产业增加值占GDP比重、文化产业增加值占GDP比重、单位GDP能耗、主要污染物排放强度指数等指标仍然是我市全面建成小康社会的短板。二是国内经济增长新旧动能转换，经济下行压力持续加大，有色金属、化工等行业市场需求低迷、产品价格大幅下跌，新能源企业窝电现象严重，工业经济效益持续下滑，实体经济亏损严重。三是产业内部结构不尽合理，传统产业竞争优势明显减弱，新的经济增长动力还未形成，第三产业发展相对滞后，非公经济总量小、比重低，生态环保刚性约束趋紧，产业结构调整、转型升级的任务十分艰巨。四是固定资产投资增速明显放缓，财政收入下降与财政支出增长的矛盾越来越突出，创新创业活力不足，培育地方财源任重道远。五是新常态下政府机关部分工作人员本领不足、本领恐慌、本领落后的问题日渐显现，面对困难和挑战不愿负责、不敢担责、不能尽责，等等。
　　**二、“十三五”经济社会发展目标和重点工作**
　　“十三五”时期，是落实“四个全面”战略布局的关键期，也是全面建成小康社会的决胜期。当前，世界新一轮科技革命和产业变革蓄势待发，我国经济发展方式加快转变，新的增长动力正在孕育形成，为我市加速技术进步与扩大对外开放带来了新契机;十八届五中全会对“十三五”时期发展作出了重大决策部署，释放了一系列重大机遇利好，为我市经济平稳较快增长和转型升级提供了新空间;省委、省政府着力打造丝绸之路经济带黄金段，深入实施“3341”项目工程，三大国家级战略平台建设加快推进，将给我们带来新的更大的发展机遇。同时，我市加快经济转型、城市转型已呈现出积极变化，为今后发展奠定了坚实基础。我们必须增强机遇意识、责任意识和紧迫意识，着力在优化结构、增强动力、化解矛盾、补齐短板上取得突破性进展。
　　根据市委《关于制定金昌市国民经济和社会发展第十三个五年规划的建议》，市政府起草了《金昌市国民经济和社会发展第十三个五年规划纲要(草案)》，提请大会审议。
　　《纲要(草案)》围绕市委《建议》提出的指导思想和遵循原则，确定了“十三五”经济社会发展的总体思路：高举中国特色社会主义伟大旗帜，以邓小平理论、“三个代表”重要思想和科学发展观为指导，全面贯彻落实党的十八大和十八届三中、四中、五中全会精神以及习近平总书记系列重要讲话精神，协调推进“五位一体”总体布局和“四个全面”战略布局，牢固树立创新、协调、绿色、开放、共享的发展理念，积极适应、把握和引领经济发展新常态，以提高经济发展质量和效益为中心，全面深化改革，加快转型升级，推进“四化”同步，促进全国资源型城市转型发展示范区、国家新型城镇化综合试点示范区、全省全面建成小康社会先行示范区“三区共建共享”，建设繁荣文明和谐现代金昌、宜居宜业宜游幸福家园，提前全面建成小康社会。
　　综合考虑“十三五”发展环境、发展基础和增长潜力，今后五年主要目标的基本考虑是：
　　——经济持续健康发展。地区生产总值保持在合理增长区间，年均增长7%以上；固定资产投资年均增长9%以上，战略性新兴产业增加值比重达到10%以上，第三产业增加值占生产总值比重达到36%以上，文化及相关产业增加值比重提高到5%以上，三次产业比调整为7.2:56.7:36.1。社会消费品零售总额年均增长8%。非公经济占生产总值比重提高到35%以上。2018年提前实现全面小康社会各项监测目标。
　　——人民生活水平普遍提高。农村贫困人口全部脱贫，城乡居民人均可支配收入到2018年比2010年翻一番，年均增长8.5%。城镇新增就业5万人，城镇登记失业率控制在4%以内。继续落实教育优先发展战略，义务教育巩固率稳定在99.88%，平均受教育年限达到10.5年以上，到2018年基本实现教育现代化。养老、医疗保险基本实现全覆盖，人均预期寿命提高到76岁以上。
　　——城镇化水平不断提高。新型城镇化稳步推进，常住人口和户籍人口城镇化率分别达到75%、72%。城乡基础设施条件进一步改善，城乡区域发展差距明显缩小。现代综合交通运输体系基本形成，能源输送能力显著增强，信息、水利基础设施和安全生产基础不断加强，基本满足经济社会发展的需要。
　　《纲要(草案)》提出以战略平台和重大举措为抓手，推动落实全面小康示范、科技创新引领、文化旅游突破、工业转型升级、农业提质增效、城乡一体融合、绿色低碳发展、城市品质提升、民生福祉改善、改革开放提速等“十大行动”，规划建设西部香草花卉示范基地、区域现代物流基地、国家重要的镍钴新材料高新技术产业基地、国家新能源示范基地、国家循环经济示范基地、全国工业固废综合利用示范基地、全国矿产资源综合利用示范基地、全省新型化工产业基地、全省高原夏菜基地、全省草食畜牧业基地等“十大产业基地”，谋划实施旅游产业、文化产业、现代流通业、信息化、有色金属及新材料产业、化工产业、能源建设、农业产业化、交通突破、水利建设、城镇基础设施建设、农村人居环境改善及民居建设与改造、精准扶贫、生态环境建设、循环经济、社会事业等“十六个项目工程包”。
　　《纲要(草案)》明确了“十三五”时期的战略重点和主要任务：
　　  (一)加快发展以文化旅游为龙头的现代服务业，推进第三产业扩规增量。顺应第三产业快速发展新态势，大力发展文化旅游、商贸物流、现代金融、信息消费等产业，促进服务业优质高效发展。着力打造“紫金花城•神秘骊靬”大景区，旅游综合收入突破40亿元，把我市打造成丝绸之路经济带重要旅游目的地。抓住华夏文明传承创新区建设机遇，主动融入丝绸之路(敦煌)国家文化博览会，实施重大文化工程，完善公共文化服务体系、文化产业体系、文化市场体系，培育年产值500万元以上文化企业10家以上，文化产业增加值年均增长25%。积极推进“互联网+”流通行动，加快铁路现代物流中心、“紫金云”大数据产业园区建设，推动现代物流业和信息产业快速发展。
　　  (二)加快工业经济转型升级，提高增长质量和效益。按照“一区多园”发展布局，大力调整工业经济结构，加快提升产业核心竞争力。围绕传统产业改造升级换代，充分利用老工业基地调整改造政策，推动传统产业与新技术、新创意相结合，全面提升设计、制造、管理水平，重塑传统产业竞争新优势。围绕建设镍钴新材料高新技术产业基地，实施优质产业链培育行动，依托技术创新不断开发技术含量高、附加值高和市场需求大的产品，促进产业价值链向中高端延伸。围绕建设全省新型化工产业基地，鼓励支持发展产品档次高、工艺技术装备领先的化工项目，促进产业上下游融合，推动化工产业向精细化转变。围绕建设国家新能源示范基地，有序扩大西坡、河清滩、金武公路三个百万千瓦级光电基地，提高风光电资源利用效率。“十三五”末，实现有色金属新材料产量80万吨以上、化工产品产量1000万吨、风电装机规模200万千瓦以上、光电装机规模500万千瓦以上、太阳能热发电装机规模20万千瓦。
　　  (三)积极发展现代农业，促进农业提质增效。实施藏粮于地、藏粮于技战略，加快转变农业发展方式，着力构建现代农业产业体系、生产体系、经营体系。巩固永昌县全省养羊大县成果，壮大金川区优质肉牛基地规模，特色优势农作物种植面积稳定在96%以上，发展现代农业示范园区10个、优势特色产业基地30个。发展示范性家庭农场90个，培育示范性农民专业合作社160个。农业科技进步贡献率达到60%，建成国家农产品质量安全市。
　　  (四)实施创新驱动，增强转型发展新动能。坚持政府推动、企业主体、人才为先，加大科技创新投入，强化“双创”平台建设，推动大众创业、万众创新，支持发展众创、众包、众扶、众筹空间，研究与开发支出占生产总值比重达到4%以上。加快有色金属新材料创新创业示范园建设，重点实施科技创新突破工程、创新载体提升工程、创新资源集聚工程、创新创业服务工程、研发投入保障工程等“五大工程”，促进科技成果资本化、产业化，科技进步贡献率达到60%以上。
　　  (五)加强基础设施建设，完善城市综合功能。大力实施“6213”交通突破行动，加快公路畅通、铁路连通、航路广通、公交惠民工程建设，构建安全高效、互联互通的立体交通网络。抢抓全省实施“6363”水利保障行动机遇，重点实施“引大济西”、灌区续建配套与节水改造、小型农田水利、人饮安全工程巩固提升、防洪减灾、水源地保护等项目工程，确保水生态安全。遵循“产城一体”、“景城一体”、“城乡一体”理念，加快公共交通、供热供气、垃圾污水处理等基础设施建设，积极推进城市地下综合管廊和“海绵城市”建设，有序推进老城区街景风貌改造，全面提升市政公用设施水平和城市形象品位。
　　  (六)统筹城乡融合发展，加快推进新型城镇化进程。加快推进国家新型城镇化综合试点、永昌县全国深化县城基础设施投融资体制改革试点和金川区双湾镇国家建制镇示范试点工作，实现4.5万左右农业转移人口和其他常住人口在城镇落户，改造2万多户6万多人居住的城镇棚户区和城中村，形成可复制、可推广的经营模式。加快“美丽乡村”建设，金川区中心城区外所有行政村建成美丽乡村，永昌县所有行政村实现人居环境改造整治全覆盖，每个乡镇建成2—3个美丽乡村示范村。持续推进“双联”行动与精准扶贫精准脱贫深度融合，2016年底基本消除绝对贫困。
　　  (七)坚持绿色发展，推动生态文明建设。以国家生态安全屏障综合试验区、循环经济示范区和绿色矿业发展示范区建设为平台，严格落实资源有偿使用和生态补偿制度，合理划定城镇、农业、生态三类空间比例，加快实施重大生态工程，农产品主产区农业空间占比高于50%，重点生态功能区生态空间占比高于50%，林地面积达到361万亩，促进人与自然和谐共生。加快建设国家低碳试点城市，全面推行清洁生产和绿色制造，提高资源循环利用率。实行最严格的环境保护制度，深入实施大气、水和土壤污染防治计划，单位生产总值能耗、主要污染物排放总量和单位生产总值二氧化碳排放量控制在省上下达指标以内，切实维护生态环境安全。
　　  (八)促进共享发展，增加民生福祉。坚持就业优先战略，大力促进创业就业。实行全民参保计划，建立更加公平更可持续的社会保障制度。以建设均衡、高质量的现代化教育为核心，实现学前教育普惠发展、义务教育均衡发展、高中教育多样发展、职业教育特色发展、成人教育开放发展、特殊教育延伸发展。加快建设健康城市，加强市、县、乡(镇)、村(社区)四级医疗卫生服务网络建设，深化公立医院改革，发展中医药事业，完善公共卫生服务体系。推进社会养老服务，加快建立覆盖城乡的新型社会救助体系。加快各类保障性住房建设，健全住房保障和供应体系，改进住房公积金提取、使用、监管机制，提高住房公积金使用效率。
　　  (九)深化改革开放，增强发展动力活力。坚持问题导向，全面推进各项改革举措，加快形成有利于创新发展、协调发展、绿色发展、开放发展、共享发展的体制机制。深化行政体制改革，稳妥推进事业单位分类改革，推进简政放权。深化农村综合改革，加快推动农村资产“确权赋能”，积极推进国有林场改革、水务改革。深化国资国企改革，大力发展混合所有制经济，加快推进企业上市步伐。深化财税金融改革，优化财政支出结构，鼓励金融机构创新和丰富金融产品。全面落实非公经济发展扶持政策，不断深化对内对外开放，主动加强与“一带一路”沿线国家和地区的合作交流。
　　  (十)推进民主法治建设，加强社会治理创新。加强法治政府建设，依法设定权力、行使权力、制约权力、监督权力，实现政府活动全面纳入法治轨道。深入推进“法治金昌”建设，全面落实“七五”普法规划，提高全民法律素质。加强精神文明建设，巩固提升全国文明城市创建成果。推进军民融合式发展，巩固双拥创建成果。完善村民自治、城市居民自治和企事业民主管理制度，扩大基层民主。加强“平安金昌”建设，推进社会治理精细化，构建全民共建共享的社会治理格局。
　　**三、2016年主要工作**
　　各位代表，今年是实施“十三五”规划的开局之年，也是全面深化改革的关键一年。做好今年的政府工作，对于实现“十三五”规划良好开局、提前全面建成小康社会意义十分重大。主要预期目标是：生产总值增长6%左右；固定资产投资增长7%左右；一般公共预算收入增长6%左右；社会消费品零售总额增长8%左右；进出口贸易总额增长5%左右；城乡居民人均可支配收入分别增长8%和10%左右；居民消费价格指数涨幅控制在3%以内；战略性新兴产业增加值占GDP比重提高1个百分点以上；科技对经济增长的贡献率提高1个百分点以上；万元GDP能耗下降1%以上；城镇登记失业率控制在4%以内；户籍人口和常住人口城镇化率分别提高2个和1个百分点以上。
　　实现上述目标，在继续用好国家适度扩大需求政策的同时，紧紧抓住影响全市经济社会发展的关键点，对接用好中央确定的宏观政策要稳、产业政策要准、微观政策要活、改革政策要实、社会政策要托底的五大政策支柱，为我市转方式调结构营造良好政策环境，重点做好六个方面的工作：
　　  (一)牢牢把握稳中求进总基调，全力推动经济稳定增长。深入实施“3341”项目工程，加快推进“十六个项目工程包”落地建设，努力促进有质量、有效益、可持续的增长。
　　保持投资稳定增长。充分发挥投资对经济增长的关键作用，积极谋划实施一批战略性、全局性、基础性项目。跟踪落实国家万亿专项建设基金支持项目，积极推进PPP模式，重点抓好兰州至张掖铁路三四线、铁路现代物流中心、铜尾矿综合利用二期、国道G570下四分至永昌及省道S212线红沙岗至下四分一级公路改造、韩家峡水库、城市应急备用水源等重大项目建设。健全完善专业化、链条化招商机制，积极支持重点企业引进战略合作者，开发镍基合金、硝基复合肥、高浓度复合肥、多品种铁合金、电线电缆等项目。加快推进“引大济西”、神雾40万吨PE、中勘新能源镍氢电池、人防应急指挥中心及疏散基地、武汉天和生物质发电及危废处理、广东富海桑田甲醇汽油、江苏机电电力管材生产加工等项目前期工作，力争早获批、早开工、早投产。
　　保障企业正常运行。强化经济运行预测监测，积极帮助企业降低制度性交易成本、财务成本、电力价格、物流成本等，重点支持金川集团公司争取有色金属及深加工、电石、PVC等行业领域获得优惠电力价格政策，为企业减负松绑。着力化解经济运行中的突出矛盾和问题，扩大新能源电力就地消纳和大用户直供电范围，帮助停产半停产企业尽快复产，抓好30万吨PVC、40万吨离子膜烧碱二期、40万吨电石、16万吨干法乙炔等建成项目投产工作，推动工业企业利润增速回升。加大银企对接力度，协调金融机构采取转贷、续贷、缓收贷款利息、延长贷款偿还期限等措施帮助生产经营困难企业渡过难关。着力打造P2B互联网融资服务平台,积极推进“助保贷”业务，“助保贷”业务贷款不低于1亿元。
　　促进居民消费升级。准确把握服务消费、信息消费、绿色消费、时尚消费、品质消费、农村消费等重点领域和方向，着力实施商业综合体、民俗体验、传统文化体验村等项目,加快培育形成经济发展新供给新动力。大力推进宽带提速降费、城市光纤入户、城区无线上网覆盖工程，积极争取开展全国农村宽带普遍服务补偿机制试点工作，促进“三网”融合，推动电商、网购、快递等快速发展。围绕实施省上提出的精准扶贫电商支持计划，积极争取阿里巴巴集团将淘宝全国农村电商平台落户金昌，加快推进永昌县省级电子商务示范县建设，改建县乡便民市场2个、改造星级农家乐20个，建设乡(村)电商服务站点40个，建成再生资源回收体系分拣中心1个，线上线下特色农产品销售量占农产品总量的15%。
　　进一步扩大对外开放。借助“一带一路”等国家战略，支持金川集团公司PVC管材项目落户白俄罗斯中白工业园，八冶建设集团公司在吉尔吉斯斯坦建设100万吨水泥项目。加快推进丝绸之路经济带甘肃黄金段重要节点城市建设，积极争取对外投资合作贷款贴息、进口贴息及中小企业国际市场开拓资金，推动民营企业“走出去”寻求发展空间，促进区域合作与发展。依托贸促、会展等平台大力推介金昌元素，推动开展经贸交流、产业对接活动。充分发挥金昌海关和出入境检验检疫局职能作用，提高贸易便利化水平。
　　  (二)加快调整优化产业结构，着力提高经济发展质量和效益。积极适应新常态下速度变化、结构优化、动力转化三大特点，加大供给侧结构性改革力度，加快形成顺应新常态、重塑新动力的发展方式。
　　做大三产。坚持把文化旅游产业作为各级政府抓现代服务业的首位工程抓紧抓好抓实，围绕建设西部香草花卉示范基地，加快推进“紫金花城•神秘骊靬”大景区建设，重点实施玫瑰谷、婚庆文化街、国家画院金昌创作基地等项目。启动实施骊靬遗址保护开发项目，抓好以骊靬文化产业园为依托的房车及自驾车营地、游客集散服务中心、骊靬影视城、骊靬文化展示中心等项目。加强与农垦企业合作发展，实施虞美人特色花卉生态观光旅游基地等项目。加快“昊客云台”专业电商平台、芙蓉公馆老年社区、老汽车站东方时代广场、尚都百汇商业广场等项目建设，扶持新城子镇油菜花观赏基地、南坝乡“枫叶印象”景观带、红山窑乡陶艺等乡村旅游项目，开工建设省道301线破城子至军马四场、省道308线何家湾至永昌道路。启动铭峰首座奥特莱斯广场，加快国芳万和城商业综合体、“印象金昌”文化创意园商业风情街建设进度，为积聚城市人气、促进三产发展创造条件。
　　做精二产。抓住结构调整窗口期，落实“中国制造2025”甘肃行动纲要和“互联网+制造”行动，加快互联网技术在企业生产、销售、管理全过程的综合集成应用，重点实施新兴产业培育扩能、传统产业提质升级、循环产业延伸拓展、“僵尸企业”清理处置为主的工业转型升级行动。扎实做好金昌有色金属新材料战略性新兴产业区域集聚发展试点工作，大力支持金川集团公司推进粉末冶金制品产业园、电池产业园、电镀产业园、电工材料产业园建设，切实加快“紫金云”大数据产业园、金川公司二选车间扩能降耗技术改造、危废资源化处置中心、高纯度锂盐提纯、超细金属及氧化物等项目建设，着力促进铜阳极泥贵金属综合回收利用、羰基镍及铂族金属原料制备技术改造、金泥电石炉净化尾气综合利用、元生工农业复合循环经济产业化等项目尽快投产。加强金武张千万千瓦级风光电基地建设前期工作，加大新能源项目争取和建设力度，积极推广分布式光伏发电，实施城区电网完善工程和35千伏及以下电网升级改造工程。
　　做优一产。围绕建设全省高原夏菜基地、全省冷凉灌区食用菌产业基地、全省草食畜牧业基地，大力实施农业提质增效行动，加速农业与资本、科技、创意、信息等要素融合，不断增强农业发展内生动力。打造规模农业，依法规范农村土地流转，推动农村土地向种养大户、家庭农场、农民合作社、龙头企业集中，着力抓好润昌源农业综合市场、和鑫源蔬菜冷链物流、昌锦源马铃薯淀粉加工、源泰黑果枸杞生产线等项目，组建集生产、加工、销售、培训、科研为一体的金昌现代农业股份集团公司，认定市级以上示范性农民合作社30家，家庭农场20家，建立股份合作社10家，促进农业工业化、管理现代化、种植科学化。打造高新农业，加大农业科技推广应用，加强高效节水示范、高标准农田等项目建设，推广膜下滴灌等高新节水技术，发展高效节水灌溉面积13万亩，建立标准化示范基地15万亩，引进农业新品种、新技术120个，农产品抽检质量合格率达到98%以上，农机化综合作业水平达到81%。打造品牌农业，着力发展壮大特色品牌农业，实施“百村万棚质量提升计划”和“舍饲养殖能力提升计划”，新建改造日光温室500座、食用菌棚500座、标准化养殖小区(场)10个，新建塑料大棚1000座，特色优势农作物种植面积占总播种面积的95%以上，香草花卉、食药赏多用蔬菜等示范面积达到5000亩。
　　  (三)全面推进改革创新，持续激发发展活力和内生动力。大力实施改革开放提速行动，加快形成有利于发展的行政体制、市场环境、产权制度、科技创新机制，着力培育新的发展动力和竞争优势。
　　深化行政管理体制改革。持续推进简政放权、放管结合、优化服务，深化行政审批制度改革，推行“三张清单一张网”管理模式，今年再取消下放一批行政审批项目，进一步激发市场活力和社会创造力。深化商事制度改革，推行市场准入负面清单管理模式，全面落实“三证合一”登记制度。在行政和参公事业单位先行开展公务用车制度改革。做好环保机构监测监察执法垂直管理工作，增强环境执法的统一性、权威性、有效性。加快整合不动产管理职能，健全市县两级不动产统一登记机构。
　　推进国资国企改革。积极支持金川集团公司全面放开有色金属深加工领域，支持八冶建设集团公司混合所有制改革试点工作。抓紧组建国有资本投资运营公司，尽快出台金化集团、金铁集团、金泥集团等市属国有企业扭亏脱困方案和“一企一策”改革方案，切实解决好企业转岗分流人员、困难职工安置工作和生活保障问题，促进困难企业尽快走出困境。加快金泥集团公司、三洋金源农牧股份公司、宇恒镍网公司等企业上市，支持金化集团公司、金铁集团公司等市属国有企业引进战略合作者通过兼并重组、股权转让等方式，发展混合所有制经济，焕发企业生机和活力。
　　深化财税体制改革。全面推进预算绩效管理工作，加强绩效评价结果应用，硬化预算刚性约束。推进事权和支出责任划分改革，建立跨年度预算平衡机制，编制三年滚动财政规划。严格政府举债，建立和完善政府债务风险预警机制，有效防范政府性债务风险。加强对财政资金分配使用、公共资源转让、公共工程建设等权力集中部门的监督管理，切实保障公众的知情权、参与权和监督权。
　　深化农村综合改革。加快推进农村集体土地使用权、土地承包经营权、宅基地和农宅、农牧业设施、集体建设用地确权工作，加快建立农村产权流转交易市场，推动农村资产“确权赋能”，县、区农村产权交易中心建成投用。深化集体林权制度配套改革，推进国有林场改革。探索筹建市水务投资公司，推进水资源管理、水权水市场、水利设施建设投融资、水利工程管理等领域改革。
　　加快推进科技创新。围绕创建省级创新型城市，深入实施创新驱动战略，大力实施科技创新引领行动，积极支持金川集团公司工程技术研究中心等科技创新平台建设，推广先进适用技术和高新技术，提升各类园区产业承接、孵化、培育能力，甘肃有色金属新材料创新创业示范园科技孵化及检测中心建成投用。深入推进企业与高等院校、科研院所技术合作交流，切实加强“人才特区”建设，加快形成集聚整合各类科技创新要素的工作机制，使创新潜力充分涌流、创新人才充分展现。深入开展“全国质量强市示范城市”创建活动，全面提升质量水平和竞争力。
　　  (四)加快推进新型城镇化建设，促进城乡区域协调发展。坚持走集约、智能、绿色、低碳、和谐的城镇化道路，大力实施城乡一体融合行动和城市品质提升行动，切实加快新型城镇化进程。
　　加快新型城镇化试点工作。稳步推进国家新型城镇化综合试点及省级新型城镇化试点和小城镇规划建设等工作，不断提高城市规划建设和管理水平，加快形成政府主导、覆盖城乡、可持续的基本公共服务体系。进一步深化户籍制度改革，健全完善居住证制度和人口信息管理制度，促进有能力在城镇稳定就业和生活的农业转移人口举家进城落户，并与城镇居民享有同等的权利和义务，实现基本公共服务常住人口全覆盖。积极组建村镇银行、民营银行、农村商业银行和农村资金互助社，支持金融机构发展供应链服务新模式，拓宽普惠金融服务领域。着力推进以满足新市民为出发点的住房制度改革，化解房地产库存，扩大有效需求，稳定房地产市场。
　　加快完善城乡规划体系。坚持区域融合与城乡一体并举,新区建设和旧区改造结合,以产业升级和城市转型为主线，突出先进、长远、超前、留白的规划理念，启动编制中心城区综合管廊建设规划，完成市域城镇体系规划、庆阳路以北、永昌路以西、新华路以南等地块及“紫金云”大数据产业园区控制性详细规划，以及永昌县“多规合一”、县域村庄整治和美丽乡村等规划。积极推行“阳光规划”，严格规划实施监督管理，不断提升规划管理精细化水平。
　　加快城乡基础设施建设。积极推进金水新区和龙首新区建设，新改建北部绿色长廊北侧等6条城市道路。抓好城市供热管网、城市供水改扩建、雨水收集利用、延安西路北侧绿化、开发区工业配水工程、开发区供热工程等项目建设。加大城镇棚户区和城中村改造力度，有序推进老旧住宅小区综合整治，加强保障性安居工程建设，推进盘旋路西南角、14区昌荣里西片、八冶公司4号区、金川集团公司3号区棚户区改造等项目。进一步扩大天然气入户覆盖范围，实施好永昌县和河西堡天然气综合利用、县城供水供热、312国道县城过境段改造等项目，公安局业务技术用房、区城乡客运站、县客运中心建成投用，完成农村公路通畅及路网改善工程100公里。
　　加快建设高效智能城管。坚持消除盲点、破解难点、突出重点、增加亮点，深入开展“五治”专项行动，加大重点区域综合整治力度。推进国家“智慧城市”试点工作，启动实施“数字城管”二期及“智慧城管”项目，全面推进城市管理网格化、精细化、数字化。坚持疏堵结合、以疏为主，着力加强建筑垃圾管理，坚决杜绝城区周边偷运乱倒建筑垃圾现象发生，全力整治城市占道经营行为，让坐商归店、游商归市，还民众一个文明、整洁的城市环境。以规范秩序、完善设施、建立长效机制为重点，加大交通违法行为整治力度，创建交通便利、出行安全、秩序井然的交通秩序。
　　  (五)切实加强生态环境保护，筑牢绿色安全发展屏障。牢固树立环保底线、生态红线“双线思维”，坚持生态立市、绿色发展、绿色惠民，大力实施绿色低碳发展行动，加快形成绿色发展方式和生活方式。
　　大力推进生态建设工程。高度重视和持续关注祁连山水源涵养林生态保护工作，积极创建国家级绿色矿业发展示范区，大力实施矿山复绿行动，争取实施北海子国家湿地公园、金昌国家沙漠公园等重点生态工程，切实抓好天然林保护、三北防护林五期、新一轮退耕还林、重点公益林管护、永昌县沙化土地封禁保护试点续建和金川河河道治理等项目，着力实施市区西部沙枣胡杨观赏林一期和高速公路枢纽区绿化等工程，完成人工造林0.9万亩，封滩育林2.1万亩，管护重点公益林178.79万亩，筑牢生态安全屏障。
　　切实加大环境保护力度。围绕建设全国工业固废综合利用示范基地和全国矿产资源综合利用示范基地，深入实施工业污染达标排放计划，完成金川集团公司镍闪速炉等系统环保集气达标治理、三洋农牧生产废水处理、易农生物公司有机肥生产等重点减排项目，确保主要污染物减排量控制在省上下达指标范围内。深入实施水污染防治行动计划，加强饮用水水源地保护，实施金川集团公司工业废水升级治理工程，加快推进永昌县、河西堡镇污水处理厂中水利用工程建设，完成市污水处理工程升级改造及中水利用工程，确保水环境安全。深入实施大气污染防治行动计划，大力落实大气污染防治“8+1”工作方案，从减排、降尘、防烟、禁烧、控车、压煤等关键环节入手，着力抓好氨氮、氮氧化物、二氧化硫、化学需氧量、可吸入颗粒物等主要污染物治理工作，确保市区空气质量持续改善。深入实施农村环境连片整治计划，加大农业面源污染防治力度，持续推进“十村示范百村整治工程”，重点实施5个建档立卡贫困村的环境连片整治，着力整治畜禽养殖污染、生活垃圾污染、生活污水等环境脏乱差问题，带动提升农村人居环境质量。
　　加快推进美丽乡村建设。按照“空间优化形态美、功能配套村容美、兴业民富生活美、生态优良环境美、乡风文明和谐美”的总体要求，加大农村集中居住点规划建设力度，建成省级美丽乡村示范村3-5个，市级美丽乡村示范村2个，每个乡镇新建、改造或扩建集中居住点1-2个，努力把广大农村建设成更有古道乡愁、更加和谐稳定、更富人文情怀的美丽乡村。
　　  (六)统筹发展各项社会事业，着力保障改善民生。坚守底线、突出重点、完善制度、引导预期，大力实施民生福祉改善行动，在完成省上下达我市惠民实事任务的基础上，继续实施民生“八大工程”。
　　实施脱贫攻坚工程。进一步促进扶贫攻坚与“双联”行动融合联动，全面落实“1+9”精准脱贫方案，坚决打好脱贫歼灭战，基本消除绝对贫困。完成红光园艺场易地扶贫搬迁一期项目建设任务。
　　实施就业创业工程。深入实施大学生就业促进计划和创业引领计划，加大有针对性的职业技能培训力度，提高劳动力素质和就业创业竞争能力，帮助失业人员和就业困难人员实现就业，新增城镇就业1.4万人以上，发放小额担保贷款1.6亿元以上，完成职业技能培训8000人，创业培训1100人，劳务品牌培训700人，输转城乡富余劳动力8万人次，实现劳务收入14亿元。
　　实施社会保障工程。加快机关事业单位养老保险制度改革，全面落实城乡低保和社会养老保险、城乡居民临时救助等保障制度，全面实施社会保险全民参保登记计划，实现社会保险“五险合一”信息系统全覆盖，城乡居民养老保险“一卡通”全覆盖，编密织牢民生安全保障网。新建各类保障性住房及棚户区改造6037套，改造农村危房2000户，新建城市社区日间照料中心3个、农村老人幸福互助院15个。
　　实施教育提升工程。加快推进基本实现教育现代化省级实验区建设，九年义务教育巩固率保持99.88%以上，高中阶段毛入学率保持99.75%以上。推进第二期学前教育三年行动计划，学前三年教育毛入园率达到92%以上。改善义务教育办学条件，完成校舍建设面积15635平方米，完成市五中新建项目一期工程。
　　实施医疗卫生服务工程。全面推进公立医院改革，统筹推进医疗、医保、医药“三医联动”，巩固完善基本药物制度。抓好市第一人民医院全科医生培训基地、市中心医院住院部综合大楼、市养老康复中心等项目建设，完成市人民医院和市第一人民医院迁建任务，市消毒供应中心、市妇幼保健院综合业务楼竣工投用。全面实施二孩生育政策，促进人口均衡发展。
　　实施文化惠民工程。坚持用中国梦和社会主义核心价值观凝聚共识、汇聚力量，巩固全国文明城市创建成果，加强文明村镇、文明单位创建活动。加快构建公共文化服务体系、文化产业体系、文化市场体系，市传媒中心、市博物馆、金川区“三馆”建成投用，进一步提高文化馆、美术馆、非遗传承中心、乡镇综合文化站建设标准，全面完成“乡村舞台”建设任务。
　　实施体育惠民工程。大力开展群众性全民健身活动，免费为市民开展国民体质监测工作，引导市民科学健身。办好中国•金昌国际青少年生存训练营活动，加快以金沙体育运动休闲园和青少年生存训练营营地为重要内容的体育产业园建设，建成乡镇及社区体育健身中心2个、“一村一场”5个。
　　实施社会治理创新工程。深入推进“平安金昌”建设，继续开展社会治理“五大专项行动”，不断加强社会治安防控体系建设，切实汲取永昌“12.30”群众集聚事件教训，进一步加强改进信访、调解和社区管理工作，推动重大社会矛盾和涉稳问题排查化解常态化，构建全民共建共享的社会治理格局。严格落实安全生产责任体系，不断提高安全生产保障水平。
　　实施食品安全战略，建立监管最严格、过程全覆盖的食品药品管理制度，建成永昌县、金川区小食品集中加工区。加强突发公共事件应急能力建设，建成县区应急救灾物资储备库，市应急指挥平台建成投用。积极创建国家防震减灾示范城市，提升防震减灾综合能力。
　　各位代表，完成“十三五”时期和今年的各项目标任务，对政府自身建设提出了新的更高要求，我们将忠实履行宪法和法律赋予的职责，严明政治纪律和政治规矩，着力打造法治政府、服务政府、责任政府、廉洁政府。
　　严格依法行政。政府机关工作人员要带头尊法学法守法用法，自觉接受人大、政协及社会各界监督，严格执行人大各项决议，以切实的举措支持人大履行好立法权。更好地运用法治思维和法治方式深化改革、促进发展、化解矛盾、维护稳定。健全依法决策机制，提高决策的法治化、规范化和科学化水平。完善政府权力行使的内外监督制约体系，深入推进电子政务建设，扩大政府信息公开范围。不断完善“三张清单一张网”，进一步提高政府服务社会经济发展的能力。
　　持续改进作风。认真贯彻落实中央“八项规定”精神和省市委各项规定，巩固扩大“三严三实”专题教育成果，扎紧篱笆、织密笼子，坚决反对形式主义、官僚主义、享乐主义、奢靡之风，推动作风建设落地生根。把督促检查贯穿于政府工作全过程，完善常态化督查工作机制，注重督查实效。落实从严治政责任，坚决整治不敢担当、吃拿卡要、慵懒散奢等突出问题，推动政府机关树立主动作为、干在实处、奋勇争先的施政新风，确保各项工作高标准推进和高质量完成。
　　提高执行能力。坚持法定职责必须为、法无授权不可为，依法履行政府职能，加强履责监管，保障重大决策、重大项目、重点工作落到实处。政府机关工作人员要树立全局意识，自觉把责任扛在肩上、把任务抓在手上，不得逃避责任、消极对待。弘扬改革创新精神，敢啃硬骨头、敢于攻坚克难，创造性地开展工作，在破除体制机制障碍、突破瓶颈短板中推进改革发展。大力开展行风政风评议，严格行政问责，坚决查处执行不力、失职渎职、弄虚作假等行为，确保政令畅通。
　　加强廉政建设。认真贯彻《中国共产党廉洁自律准则》和《中国共产党纪律处分条例》，严格落实党风廉政建设责任制，强化守纪律、讲规矩要求，加强监察和审计工作，严肃查处贪污腐败等违法违纪行为，做到干部清正、政府清廉、政治清明。严格执行招投标、项目审批、公共资源交易、国有资产处置等规定，有效遏制工程违法发包、转包、分包及挂靠等行为，依法规范招投标活动。强化政府投资项目监管，严控工程建设规模标准，坚决避免重复建设和超预算、超标准建设行为，不准盲目铺摊子、上项目。
　　各位代表，做好“十三五”和今年的各项工作任务繁重、责任重大，让我们在市委的坚强领导下，在爬坡过坎中攻坚克难，在转型发展中止滑稳增，在创新创业中再创辉煌，为建设繁荣文明和谐现代金昌、宜居宜业宜游幸福家园而努力奋斗！
